# Comparing Texture Analysis of Apparent Diffusion Coefficient MRI in Hepatocellular Adenoma and Hepatocellular Carcinoma

**DOI:** 10.7759/cureus.51443

**Published:** 2024-01-01

**Authors:** Ayoob Dinar Abdullah, Behzad Amanpour-Gharaei, Mohssen Nassiri Toosi, Sina Delazar, Hamidraza Saligheh Rad, Arvin Arian

**Affiliations:** 1 Technology of Radiology and Radiotherapy, Tehran University of Medical Sciences, Tehran, IRN; 2 Cancer Biology Research Center, Cancer Institute, Tehran University of Medical Sciences, Tehran, IRN; 3 Hepatology, Tehran University of Medical Sciences, Tehran, IRN; 4 Advanced Diagnostic and Interventional Radiology Research Center, Imam Khomeini Hospital, Tehran University of Medical Sciences, Tehran, IRN; 5 Medical Physics and Biomedical Engineering, Tehran University of Medical Sciences, Tehran, IRN; 6 Radiology, Cancer Institute, Tehran University of Medical Sciences, Tehran, IRN

**Keywords:** magnetic resonance imaging, texture analysis, differential diagnosis, hepatocellular carcinoma, hepatocellular adenoma

## Abstract

Aim: This study aimed to assess the effectiveness of using MRI-apparent diffusion coefficient (ADC) map-driven radiomics to differentiate between hepatocellular adenoma (HCA) and hepatocellular carcinoma (HCC) features.

Materials and methods: The study involved 55 patients with liver tumors (20 with HCA and 35 with HCC), featuring 106 lesions equally distributed between hepatic carcinoma and hepatic adenoma who underwent texture analysis on ADC map MR images. The analysis identified several imaging features that significantly differed between the HCA and HCC groups. Four classification models were compared for distinguishing HCA from HCC including linear support vector machine (linear-SVM), radial basis function SVM (RBF-SVM), random forest (RF), and k-nearest neighbor (KNN).

Results: The k-nearest neighbor (KNN) classifier displayed the top accuracy (0.89) and specificity (0.90). Linear-SVM and KNN classifiers showcased the leading sensitivity (0.88) for both, with the KNN classifier achieving the highest precision (0.9). In comparison, the conventional interpretation had lower sensitivity (70.1%) and specificity (77.9%).

Conclusion: The study found that utilizing ADC maps for texture analysis in MR images is a viable method to differentiate HCA from HCC, yielding promising results in identified texture features.

## Introduction

Hepatocellular adenoma (HCA) is a rare benign liver tumor primarily associated with steroid use, particularly oral contraceptives, and it is more commonly found in young women [1]. However, there has been a recent increase in HCA cases among males and those without steroid exposure, possibly due to the inflammatory subtype of HCA related to obesity and alcohol use [2]. Proper management is crucial for HCA lesions due to the potential complications, including hemorrhage and transformation into hepatocellular carcinoma (HCC) [3]. HCC is the primary malignant liver tumor that originates from hepatocytes. Early detection of HCC is crucial for effective treatment; however, the majority of cases are diagnosed at advanced stages, resulting in poor prognosis and low five-year survival rates of 2.5% [4].

While the majority of HCC cases occur in individuals with liver cirrhosis, around 20% of cases develop in non-cirrhotic patients and are typically diagnosed at advanced stages [5]. Guidelines suggest utilizing fat-sensitive imaging techniques (e.g., contrast-enhanced ultrasonography, CT, MRI) with contrast agents to detect HCA in liver lesions. These methods aid in accurate diagnosis and management by identifying specific components. They are also used to assess HCC and provide additional insights [6,7]. However, distinguishing between HCA and HCC presents a difficulty for radiologists and clinicians due to shared histopathological and imaging features. Consequently, the use of advanced imaging techniques and radiomic analysis becomes crucial to enhance accuracy in distinguishing between HCA and HCC [8,9]. Accurate diagnosis is crucial for managing HCA and HCC. Radiomics, a diagnostic tool, extracts high-throughput features from imaging across modalities, enhancing diagnostic accuracy and enabling personalized treatment strategies for HCA and HCC [6,10-12].

Recent studies have prioritized analyzing radiomic features to distinguish liver lesions, especially HCC with its high prevalence and poor prognosis [13,14]. Limited research has been conducted on using radiomic feature analysis to differentiate HCA and HCC using CT and MRI imaging features [15,16]. However, further investigation is required to establish the effectiveness of this method and gather data for future assessments in this emerging field. This study aimed to use apparent diffusion coefficient (ADC) MRI-driven radiomics features to differentiate between HCA and HCC. The findings expand our understanding of using radiomics to distinguish between HCA and HCC liver lesions, offering a less invasive and operator-independent approach.

## Materials and methods

This prospective cross-sectional study was conducted at Imam Khomeini Hospital Complex, Tehran University of Medical Sciences, Iran, from 2020 to 2022. This study aimed to recruit patients diagnosed with HCA or HCC. Patients with clinical history and suspicious imaging features indicative of HCA or HCC were included. The final diagnosis was confirmed through histology. MRI examinations were performed for liver evaluation in enrolled patients, while those with contraindications were excluded. The study included 55 patients (27 males, 28 females). Among the 106 confirmed hepatic tumors, an equal distribution of 53 tumors each was observed for both HCC and HCA, utilizing a 3T GE healthcare whole-body system for imaging.

MRI acquisition

**Figure 1:**
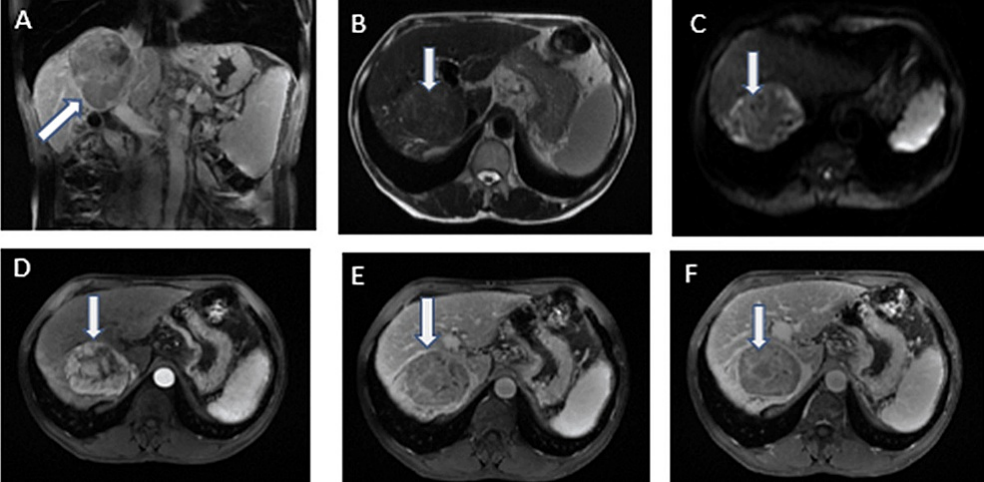
Hepatocellular carcinoma in a 66-year-old male. (A) Axial T1-weighted 3D AX GRE image shows hypointense lesion (arrow) in segment 8, (B) axial T2-weighted AX SSFSE BH image shows hyperintense lesion (arrow), (C) diffusion-weighted images show that the lesion remains hyperintense, (D) the lesion (arrow) is hypervascular on arterial phase with (E) washout on portal-venous phase, and (F) on hepatobiliary phase the nodule (arrow) is hypointense with surrounding liver parenchyma. 3D AX GRE: three-dimensional axial gradient; SSFSE BH: single-shot fast spin echo black blood

**Figure 2:**
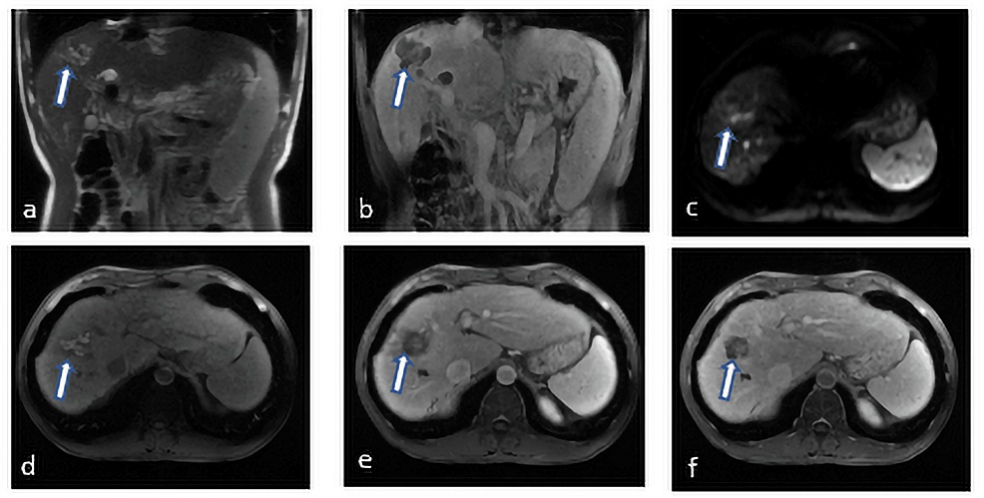
Hepatocellular adenoma in a 47-year-old female. (A) Coronal T2-weighted SSFSE BH image shows hyperintense lesion (arrow) segment 8, (B) coronal T1-weighted 3D AX GRE image shows hypointensity lesion (arrow), (C) diffusion-weighted images show that the lesion is hyperintense (arrow), (D) the lesion (arrow) is hypervascular on arterial phase (E) with washout on portal-venous phase, and (F) on hepatobiliary phase the nodule (arrow) is hypointense with surrounding liver parenchyma. 3D AX GRE: three-dimensional axial gradient; SSFSE BH: single-shot fast spin echo black blood

All patients were examined with a 3T whole-body system (GE Healthcare) in the standard supine position using an eight-channel dedicated body coil. Three-dimensional axial gradient echo T1-weighted image (3D AX GRE T1) time of repetition (TR)=8.6 ms, TE=4.7 ms, and axial single-shot fast spin echo black blood T2-weighted image (AX SSFSE BH T2WI) (TR=5600 ms, TE=57 ms) were obtained. AX 3D dual echo BH in-phase/out-of-phase (in/opp) (diffusion-weighted imaging/apparent diffusion coefficient {DWI/ADC} value: 10-3 mm^2^/s) was performed with a spin echo-echo planar imaging (SE-EPI) sequence with two b values (0 and 1000 s/mm^2^) in three orthogonal directions. The imaging parameters were as follows: TR=3300 ms, TE=94 ms, flip angle=90°, layer thickness=5 mm, matrix=128×128, and field of view (FOV)=320×320 mm. Following DWI, dynamic enhancement contrast-MRI (DEC-MRI) was performed with a three-dimensional axial LAVA dynamic (3D AX LAVA DYN) sequence (TR=4.62 ms, TE=1.75 ms, layer thickness=1.5 mm, interlayer spacing=0, FOV=360×360 mm, and matrix=384×320) before and five times after the injection of 0.1 mmol/kg gadopentetate dimeglumine (GE Healthcare). Subsequently, six-phase DEC T1-weighted image (DEC-T1W) images were acquired (Figures 1A-1F, 2A-2F).

ROI segmentation

An experienced radiologist with 16 years of experience in liver radiology performs region of interest (ROI) segmentation on the ADC map. Care is taken to include the entire tumor volume while avoiding adjacent normal liver tissue or artifacts.

Texture analysis

The texture analysis describes the process of extracting radiomic features from segmented regions of interest (ROIs) using a custom-developed algorithm. The gray-level values of images were normalized by subtracting the mean value and dividing by the standard deviation. As suggested by radiomics experts, the gray-level values were additionally scaled by a factor of 100 and a shift of 300 to avoid negative values. PyRadiomics, an open-source Python library, was utilized to extract a total of 102 radiomic features, including first-order intensity statistics features and texture features. These features capture different aspects of tumor heterogeneity, such as intensity fluctuation, spatial patterns, and voxel interactions.

Feature selection algorithms and classification

Three feature selection algorithms and four classifiers from the "scikit-learn" Python library were combined (all possible combinations) to differentiate between benign HCA and malignant HCC tumors. The feature selection algorithms employed were as follows: recursive feature elimination (RFE) algorithm [17], sequential feature selector [18], and feature selection based on the k highest scores [19].

In this study, different classifiers were employed for distinguishing between benign HCA and HCC tumors, including support vector classifier with "linear" or "radial basis function (RBF)" kernels [20]. Random forest classifier, 10 to 200 number of trees were tested to find the best number of trees [21]. K-nearest neighbors classifier (KNN), various numbers of neighbors, ranging from three to 15, were evaluated to identify the optimal value [22]. Grid search was utilized to find the best model and parameters, while four-fold cross-validation was performed to assess the models' performance. ROC curves were available for every classifier.

The texture features obtained from the analysis were subjected to statistical analysis to assess their effectiveness in distinguishing between HCA and HCC. Descriptive statistics, including mean, median, standard deviation, and range, were calculated for each texture feature. Univariate analysis, employing either the Wilcoxon p-value test or Student's t-test, was conducted to compare the texture features between the HCA and HCC groups. Receiver operating characteristic (ROC) curve analysis was performed to evaluate the diagnostic performance of the selected texture features. The sensitivity, specificity, and area under the curve (AUC) were determined from the ROC analysis.

## Results

A total of 102 features were extracted from the MRI assessments performed in the study. The comparison of these features between the two study groups showed statistical differences in 22 features based on the t-test and 45 features based on the Mann-Whitney U test. HCA was present in 48 images (102 features). Also, HCC was present in 51 images (102 features).

Out of the 102 extracted features, a total of 10 features for linear-SVM, 30 features for RBF-SVM, five features for RF, and 10 features for KNN. Among the four classifiers employed to analyze the radiomic data, the KNN exhibited the highest accuracy (0.89) and specificity (0.90). Also, the linear-SVM and KNN demonstrated the highest sensitivity of 0.88 for both, while the KNN classifier achieved the highest precision of 0.9 (Table 1).

**Table 1:** Results of the classification of HCA and HCC tumors. The first row shows the feature selection algorithm for each classifier and the number of selected features in parentheses. SVM: support vector machine; RBF: radial basis function; KNN: k-nearest neighbors

Variable	Linear-SVM	RBF-SVM	Random forest (n estimators=50)	KNN (n neighbors=3)
Accuracy	0.837	0.807	0.838	0.89
Sensitivity	0.883	0.823	0.843	0.883
Specificity	0.795	0.795	0.835	0.9
Precision	0.83	0.821	0.848	0.906

The AUC serves as a measure of the model's classification performance. A higher AUC value, closer to one, indicates superior performance in distinguishing between positive and negative classes (Table 2).

**Table 2:** Area under the curve for SVM (linear), SVM (RBF), RF, and KNN. SVM: support vector machine; RBF: radial basis function; RF: random forest; KNN: k-nearest neighbors; SE: standard error

Variables	Area	SE	Sig.	Asymptotic 95% confidence interval		Sensitivity (%)	Specificity (%)
				Lower	Upper		
SVM (linear)	0.87	0.13	0.05	0.680	0.920	85.89	81.25
SVM (RBF)	0.84	0.09	0.05	0.720	0.920	82.21	79.16
Random forest (RF)	0.87	0.13	0.05	0.640	0.960	80.12	85.41
K-nearest neighbors (KNN)	0.90	0.11	0.05	0.720	0.960	83.44	89.58

Comparisons of mean and SD of 10 features classifiers extracted from apparent diffusion coefficient (ADC) images between HCA and HCC groups are shown in Table 3. These findings suggest that the analyzed features exhibit variations between HCA and HCC, which may have diagnostic or prognostic implications in distinguishing between these two tumor types.

**Table 3:** Comparisons of mean and SD of 10 features classifiers extracted from apparent diffusion coefficient (ADC) images between HCA and HCC groups (p<0.05). HCA: hepatocellular adenoma; HCC: hepatocellular carcinoma; SD: standard deviation; glcm: gray-level co-occurrence matrix; gldm: gray-level dependence matrix; glrlm: gray-level run length matrix; glszm: gray-level size zone matrix

S.n.	Feature name	Tumor type	Mean±SD	p-Value
1	Original first order 90 percentile	HCA	691.3±1157.3	0.036
		HCC	307.9±567.9	
2	Original first-order root mean squared	HCA	550.1±861.2	0.036
		HCC	255.08±485.1	
3	Original first-order uniformity	HCA	0.290±0.208	0.0058
		HCC	0.411±0.217	
4	Original glcm maximum probability	HCA	0.256±0.230	0.00056
		HCC	0.426±0.243	
5	Original gldm dependence non-uniformity	HCA	41.81±73.81	0.011
		HCC	161.26±308.60	
6	Original gldm gray-level non-uniformity	HCA	42.41±85.58	0.002
		HCC	254.61±465.15	
7	Original glrlm gray-level non-uniformity	HCA	20.75±47.05	0.016
		HCC	50.27±69.67	
8	Original glrlm run entropy	HCA	0.663±0.227	0.000033
		HCC	0.414±0.224	
9	Original glszm large area emphasis	HCA	19.48±16.12	0.000013
		HCC	38.90±17.69	
10	Original glszm large area high gray-level emphasis	HCA	3111.47±6568.08	0.024
		HCC	108065.53±314848.70	

The diagnostic performance of MRI in distinguishing between HCA and HCC was assessed using the ROC curve. The ROC curve provided valuable information, with an area under the curve (AUC) of 0.860 (95% confidence interval: 0.783-0.936, p<0.001). In terms of discrimination between HCA and HCC, the sensitivity and specificity were determined to be 70.1% and 77.9%, respectively, as depicted in Table 4. The discrimination between HCA and HCC using the three-dimensional axial LAVA dynamic (3D AX LAVA DYN) test revealed AUC, 95% CI, p-value, sensitivity, and specificity (Table 5).

**Table 4:** Area under the curve of MRI finding. SE: standard error

Area	SE	Sig.	Asymptotic 95% confidence interval		Sensitivity (%)	Specificity (%)
			Lower	Upper		
0.689	0.052	0.001	0.587	0.792	70.1	77.9

**Table 5:** Areas under the curve of MRI findings according to 3D AX LAVA DYN. 3D AX LAVA DYN: three-dimensional imaging, axial orientation dynamic imaging; SE: standard error

Variables	Area	SE	Sig.	Asymptotic 95% confidence interval		Sensitivity (%)	Specificity (%)
				Lower	Upper		
Arterial	0.496	0.056	0.940	0.385	0.606	9.4	9.0
Venous	0.398	0.055	0.070	0.290	0.506	72.5	64.2
Delay	0.821	0.041	0.000	0.740	0.902	81	64.2

## Discussion

Oncology has recently focused on using texture analysis (TA) in various imaging modalities like X-ray, ultrasound, CT, MRI, and PET. TA has been applied to investigate the connection between textural parameters and tumor pathology data [23]. Holli et al. used textural metrics from breast MRI to differentiate between invasive lobular carcinoma and invasive ductal carcinoma [24]. Ba-Ssalamah et al. utilized contrast-enhanced CT texture parameters to distinguish between different types of gastric tumors [25]. Georgiadis et al. demonstrated the importance of texture analysis (TA) in brain MRI for distinguishing between metastases, gliomas, and meningiomas [26]. These studies highlight the strong correlation between textural features and pathogenic information.

The study concluded that texture analysis based on ADC MR imaging is a reliable method for distinguishing between HCA and HCC in the liver. The most significant results were observed when analyzing ADC map images, which showed distinct TA features between HCA and HCC tumors. These images achieved high sensitivity, specificity, precision, and diagnostic accuracy in accurately classifying HCA and HCC. The findings are consistent with another study by Stocker et al. that demonstrated the effectiveness of dimension texture analysis (2DTA) in differentiating malignant and benign hepatocellular tumors in the non-cirrhotic liver [27]. The study also reported the sensitivity and specificity when using TA features and traditional radiological interpretation.

Malignant tumors generally exhibit higher cellularity, leading to lower ADC values in diffusion-weighted imaging (DWI) compared to benign tumors. ADC values can be influenced by various factors, such as vendor, hardware, sequence, and approach [28]. Lee et al. demonstrated that DWI with an ADC map is a quantitative imaging method that is minimally affected by variations in gain factors, and several parameters derived from ADC showed significant differences between benign and malignant soft-tissue tumors [29]. Another study found significant differences in first-order-based ADC parameters between intermediate and high-grade sarcoma patients using 1.5 T MRI [30]. Additionally, a previous study on myxoid soft-tissue tumors found a significant difference in kurtosis of ADC between benign and malignant cases [31]. While the ADC is an important quantitative parameter in diffusion-weighted imaging (DWI), qualitative analysis of DWI with low and high b values is also valuable. DWI with a high b value does not simply serve as an inverted image of the ADC but also provides distinct information. It's worth noting that not all regions with low ADC values indicate high cellularity; they could instead be attributed to fatty components or T2 blackouts caused by hematoma [32].

Our study utilized ADC map images to identify multiple TA features, including 102 features, and found statistically significant differences between HCA and HCC. The classification of these features using linear-SVM, RBF-SVM, random forest, and k-nearest neighbors (KNN) revealed that the KNN model of TA features performed better in distinguishing HCA from HCC lesions. Based on these results, the k-nearest neighbors (KNN) classifier exhibited the highest accuracy (0.89) and specificity (0.90). The reasons behind KNN's strong performance can be attributed to its ability to capture local patterns, its non-parametric nature that allows it to handle complex decision boundaries, and its reliance on nearest neighbors for classification. However, it's important to note that KNN also has some limitations, such as sensitivity to the choice of the number of neighbors (k) and computational inefficiency on large datasets, which should be considered when selecting an appropriate classifier for a specific task. In a previous study, a logistic regression model utilizing TA features from arterial-phase images achieved an accuracy of 84.5% (sensitivity 84.1%, specificity 84.9%) for HCC diagnosis. The logistic regression model demonstrated higher accuracy compared to other models reported in the literature [33-37].

Additionally, results from a study by Książek et al. indicated that their proposed model achieved the highest accuracy in detecting HCC [38]. The study observed significant differences between HCA and HCC in certain MRI sequences, while some sequences showed no significant differences [39]. Dynamic MRI proved effective in distinguishing between the two conditions, with an AUC of 0.860, indicating good discrimination. Sensitivity and specificity were 70.1% and 77.9%, respectively. Our study had limitations, including the absence of a matched comparison between DWI images and surgical samples. Findings may be specific to the scan manufacturer and magnet strength used in the study. Additionally, further research is needed to determine if our findings can be applied to different scan manufacturers or magnet strengths.

## Conclusions

To conclude, utilizing texture analysis of ADC MR images presents a viable and objective approach for distinguishing HCA from HCC in the liver. Through our analysis, we identified 10 imaging features that exhibited the potential to differentiate between these two groups. These findings highlight the promising utility of texture analysis as a tool for improving the differentiation of HCA and HCC lesions.
